# Establishment and characterization of TRI-LC21: a novel patient-derived cell line of SMARCA4-deficient undifferentiated thoracic tumor

**DOI:** 10.1007/s13577-026-01418-9

**Published:** 2026-07-09

**Authors:** Shasha Li, Hao Luo, Dongsheng Wu, Yin Ku, Nanzhi Luo, Zhipeng Gong, Wenjing Zhou, Xinyi Xie, Guanyu Zhou, Yaohui Chen, Lunxu Liu

**Affiliations:** Department of Thoracic Surgery and Institute of Thoracic Oncology , West China Hospital, Sichuan University, Chengdu, 610041 China

**Keywords:** SMARCA4-deficient thoracic tumor, SMARCA4-UT, Patient-derived cell line, Lung cancer, Tumor model

## Abstract

SMARCA4-deficient thoracic undifferentiated tumor is a rare and highly aggressive subtype of lung cancer defined in the 2021 World Health Organization classification. Its scarcity and highly undifferentiated histology limit the availability of representative in vitro models and hinder therapeutic development. In this study, we established a novel cell line, TRI-LC21, from the primary tumor of a patient with lung cancer showing pathological features consistent with this entity. TRI-LC21 exhibited stable proliferation, strong colony-forming ability, and robust tumorigenicity in subcutaneous xenograft models. Xenograft tumors recapitulated the histological features and immunophenotype of the original tumor. Cell line authenticity was confirmed by short tandem repeat profiling, and mycoplasma contamination was excluded. Whole-exome sequencing revealed SMARCA4 loss accompanied by co-mutations in *TP53*, *STK11*, *RBM10*, and *ARHGAP35*. Transcriptome analysis demonstrated decreased expression of epithelial-associated genes and increased expression of stemness- and plasticity-related programs, consistent with an undifferentiated phenotype. Collectively, TRI-LC21 faithfully recapitulates the histological, immunophenotypic, and molecular characteristics of this tumor type and provides a valuable in vitro model for mechanistic investigation and preclinical studies.

**Supplementary Information:**

The online version contains supplementary material available at 10.1007/s13577-026-01418-9.

## Introduction

The SWI/SNF chromatin remodeling complex plays a critical role in the regulation of gene expression, and mutations in its subunits are observed in approximately 20% of human tumors [1, 2]. SMARCA4 (also known as BRG1), a key catalytic subunit of this complex, is essential for maintaining chromatin stability and cellular differentiation [3–6]. Loss-of-function alterations in SMARCA4 can drive the development of highly aggressive and undifferentiated tumors [7, 8]. Thoracic SMARCA4-deficient undifferentiated tumor (SMARCA4-UT) represents a distinct and highly lethal subtype of lung cancer [9].

This tumor was initially described as “SMARCA4-deficient thoracic sarcoma” [10]. Subsequent molecular studies revealed that its genomic features are closely related to smoking-associated non-small cell lung cancer (NSCLC) [11, 12], suggesting that most cases originate from dedifferentiated or undifferentiated tumors of pulmonary epithelial origin. With the increasing understanding of its molecular characteristics and biological nature, the World Health Organization (WHO) reclassified this entity as a distinct tumor type in the 2021 Classification of Thoracic Tumors, noting that although SMARCA4 deficiency can also occur in conventional NSCLC, SMARCA4-UT exhibits distinct histological, immunophenotypic, and biological features [9, 13, 14]. Its defining features include loss of SMARCA4 protein expression and undifferentiated or rhabdoid morphology, typically accompanied by lack of lineage-specific markers such as TTF-1 and p40, as well as frequent expression of stemness-associated markers, including SOX2 and SALL4 [12, 15, 16]. Clinically, SMARCA4-UT is associated with an extremely poor prognosis, with a median overall survival of approximately 4–7 months and generally responds poorly to conventional cytotoxic chemotherapy [9–11, 17].

SMARCA4-UT is a rare and recently defined malignancy, with only several hundred cases reported in the literature to date [9, 15, 18, 19]. It predominantly affects young to middle-aged male smokers and is frequently diagnosed at an advanced stage [18]. Due to its undifferentiated morphology, it is often misdiagnosed as poorly differentiated non-small cell lung cancer, sarcoma, or lymphoma, which further complicates clinical management and limits systematic investigation [20–22]. At present, preclinical models for SMARCA4-UT remain limited.

In recent years, a subset of previously established lung cancer cell lines has been suggested to represent SMARCA4-UT following re-evaluation. In 2023, Alberto M. Arenas et al. first suggested that the NCI-H522 cell line, originally classified as lung adenocarcinoma, may serve as a SMARCA4-UT model based on comprehensive pathological and molecular analyses [23]. Building on this concept, Jin Ng et al. subsequently reported in 2024 that three classical SCLC-Y (YAP1-expressing small cell lung cancer) cell lines (DMS114, SBC5, and H841), identified through multi-omics and pathological re-evaluation, also exhibit features consistent with SMARCA4-UT [24]. In addition, computational analysis using the DepMap/Celligner platform (https://depmap.org/portal/celligner/) identified four cell lines (H196, H841, SW1271, and DMS114) with transcriptomic similarity to SMARCA4-UT; however, only H841 and DMS114 have been experimentally validated [24], whereas the remaining lines lack systematic pathological, immunophenotypic, and genomic characterization. Collectively, currently available SMARCA4-UT cell models remain limited (including H522, DMS114, SBC5, and H841), and all have been identified through retrospective reclassification of previously established lung cancer cell lines rather than prospective derivation from primary tumors. This difference in origin and establishment context may limit their ability to fully recapitulate the biological characteristics and evolutionary trajectories of SMARCA4-UT. Therefore, well-defined, systematically characterized patient-derived in vitro models remain scarce.

In this study, we established and comprehensively characterized a novel SMARCA4-UT cell line, TRI-LC21, derived directly from the primary tumor of a patient with pathological features consistent with SMARCA4-UT. As a patient-derived model, TRI-LC21 complements existing reclassified cell lines and provides a valuable platform for investigating the pathogenesis and exploring potential therapeutic strategies for this aggressive tumor subtype.

## Materials and methods

### Tumor material

The clinical specimen was obtained from a 72-year-old male patient with lung cancer, whose tumor was located in the right lower lobe. The patient underwent video-assisted thoracoscopic surgery (VATS), including right lower lobectomy, lymph node dissection, and pleural adhesiolysis. Fresh tumor specimens collected during the surgical procedure were used for cell line establishment.

### Primary cell preparation and cell culture

Fresh lung tumor tissue obtained from the patient was minced into approximately 1–2 mm^3^ fragments under sterile conditions. The tissue fragments were digested at 37 °C for 1 h in a digestion solution containing type I collagenase (1.25 mg/mL; Gibco, 17100017) and type IV collagenase (1.25 mg/mL; Gibco, 17104019). The resulting cell suspension was filtered through a 70-μm cell strainer to remove undigested tissue fragments. The collected cells were cultured in DMEM (Gibco, 11320033) supplemented with 10% fetal bovine serum and 100 U/mL penicillin–streptomycin at 37 °C in a humidified incubator with 5% CO₂. During the culture process, fibroblasts and other non-tumor cells were gradually eliminated using a combination of mechanical scraping, differential adhesion, and selective trypsinization. Cells were passaged at a ratio of 1:3 when reaching approximately 80–90% confluence. After more than 30 consecutive passages with stable morphology and growth characteristics, the cell line was designated as TRI-LC21. The cell line has been deposited in the China Center for Type Culture Collection (CCTCC; accession number: C202581). Researchers interested in obtaining TRI-LC21 may contact CCTCC for information regarding access and application procedures, or contact the corresponding authors for further information.

### Cell lines and authentication

The TRI-LC21 cell line was established from primary tumor tissue as described above. Cell line identity was confirmed by short tandem repeat (STR) profiling, and routine mycoplasma testing verified the absence of contamination. The human bronchial epithelial cell line BEAS-2B was obtained from an in-house stock in our laboratory. According to laboratory records, the cell line was originally obtained from a commercial source. Cells were used within 10 passages after thawing. The identity of the cell line was further supported by STR profiling. The STR profile of BEAS-2B cells is provided in Supplementary Fig.S1.

### Mycoplasma detection

To detect mycoplasma contamination, culture supernatants were collected from TRI-LC21 cells after 72 h of incubation. Mycoplasma detection was performed using the EZ-Detect™ Mycoplasma Detection Kit (Ubigene, Cat# YK-DP-20). The amplified DNA fragments were visualized under ultraviolet (UV) illumination.

### STR profiling

STR analysis was performed to authenticate the TRI-LC21 cell line and confirm its origin from the matched patient tumor tissue. Genomic DNA was extracted using the Axygen Genomic DNA Extraction Kit following the manufacturer’s instructions. A 21-locus STR amplification panel, including the Amelogenin locus for sex determination, was applied. PCR products were analyzed on an ABI 3730XL Genetic Analyzer (Applied Biosystems). STR profiles of TRI-LC21 and the corresponding tumor specimen were compared to verify genetic consistency. Cell line authentication was further conducted using the DSMZ Cell Line Authentication Tool, which incorporates reference STR datasets from ATCC, DSMZ, JCRB, and RIKEN (2455 authenticated cell lines). For BEAS-2B cells, a simplified STR analysis based on selected loci was performed to support cell line identity.

### Histopathological evaluation

Formalin-fixed paraffin-embedded (FFPE) tumor tissue Sects. (4 μm thick) were prepared for histopathological assessment. Hematoxylin and eosin (H&E) staining was performed to evaluate the morphological features. Immunohistochemical (IHC) staining was carried out using a BOND RX Fully Automated Research Stainer (Leica), an automated immunostaining system. After staining, the sections were coverslipped and examined under a light microscope. The primary antibodies used included BRG1 (Abcam, ab110641), TTF-1 (Abcam, ab76013), SMARCA2 (Abcam, ab240648), SMARCB1 (Abcam, ab192864), SOX2 (Abcam, ab97959), p40 (Abcam, ab7753), CD34 (Abcam, ab81289), SALL4 (Abcam, ab29112), Vimentin (Cell Signaling Technology, #5741), CD56 (Proteintech, 14,255–1-AP), and Ki-67 (Abcam, ab16667).

### Cell growth analysis

Cell proliferation was evaluated using the Cell Counting Kit-8 (CCK-8; Beyotime, Cat# C0039). Briefly, cells were seeded into 96-well plates at a density of 3 × 10^3^ cells per well and cultured at 37 °C in a humidified incubator with 5% CO₂ for 0, 24, 48, 72, and 96 h. At the indicated time points, 100 μL of DMEM containing 10 μL of CCK-8 solution was added to each well, followed by incubation for an additional 1 h at 37°C. The absorbance at 450 nm was measured using a microplate reader (Epoch 2, BioTek). Cell growth curves were plotted using GraphPad Prism software.

### Colony-forming assay

Cells were seeded into 6-well plates at densities of 1000, 1500, and 2000 cells per well and cultured for 14 days under standard conditions. Colonies were fixed with 4% paraformaldehyde for 30 min and stained with 0.1% crystal violet for 10–20 min at room temperature. After washing with PBS and air drying, images were captured using a gel imaging system (ChemiDoc MP, Bio-Rad, USA). Colony number and area were quantified using ImageJ software.

### Tumorigenesis assessment in nude mice

Log-phase TRI-LC21 cells were harvested after digestion with 0.25% trypsin. A total of 2 × 10⁶ TRI-LC21 cells were subcutaneously injected into the left flank of 5-week-old female athymic nude mice (n = 5). Tumor growth was monitored by measuring tumor size with calipers every 7 days. Tumor volume was calculated using the formula: Volume = (length × width^2^)/2. Tumor growth curves were generated using GraphPad Prism software.

### Chromosome spread preparation and Giemsa staining

Log-phase TRI-LC21 cells were treated with 0.2 μg/mL colchicine (GLPBIO, Cat# GC40664) at 37 °C for 2 h, followed by hypotonic treatment with 0.075 M KCl at room temperature for 20 min. The cells were fixed in a methanol and acetic acid (3:1, v/v) mixture for 15 min, followed by three washes with the same fixative solution. The cell suspension was dropped onto cold microscope slides and stained with Giemsa (Solarbio, Cat# G1010) for 5 min. Well-dispersed and moderately stained metaphase chromosome spreads were observed under the microscope.

### Whole-exome sequencing (WES)

Genomic DNA was extracted and subjected to whole-exome enrichment using the Agilent SureSelect Human All Exon V6 Kit (Agilent, USA, Cat# 5190–8864). The captured libraries were subsequently amplified using high-fidelity polymerase. The final libraries were quantified by qPCR to ensure an effective concentration of 1.5 nM. Paired-end 150 bp sequencing was then performed on the Illumina platform (PE150).

### RNA sequencing analysis

To explore the transcriptomic changes in TRI-LC21 cells, the normal bronchial epithelial cell line BEAS-2B was used as a control. Biological replicates were included for each group (*n* = 3 per group). Total RNA was extracted, and RNA sequencing (RNA-seq) was performed using the Novaseq-PE150 platform (Novogene). Library preparation followed the standard protocol provided by Novogene.

## Results

### Creation and validation of the TRI-LC21 cell line

Fresh tumor tissue was obtained from the primary lung lesion of a 72-year-old male patient and subjected to primary culture to establish a novel SMARCA4-UT cell line. The tumor was located in the right lower lobe of the lung. Postoperative pathological examination revealed a malignant tumor with extensive necrosis. Immunohistochemical analysis demonstrated a complete loss of SMARCA4 expression in tumor cells. Epithelial and neuroendocrine markers, including CK7, TTF-1, Napsin A, CK5/6, p63, p40, CD56, chromogranin A (CgA), WT-1, and calretinin (CR), were all negative. In contrast, EMA, pan-cytokeratin (PCK), and synaptophysin (Syn) showed only focal positivity. SMARCB1 expression was retained, and the Ki-67 labeling index was approximately 50%. Based on the combined morphological and immunophenotypic features, the tumor was diagnosed as a malignant neoplasm with loss of BRG1 protein expression, consistent with SMARCA4-UT.

**Fig. 1:**
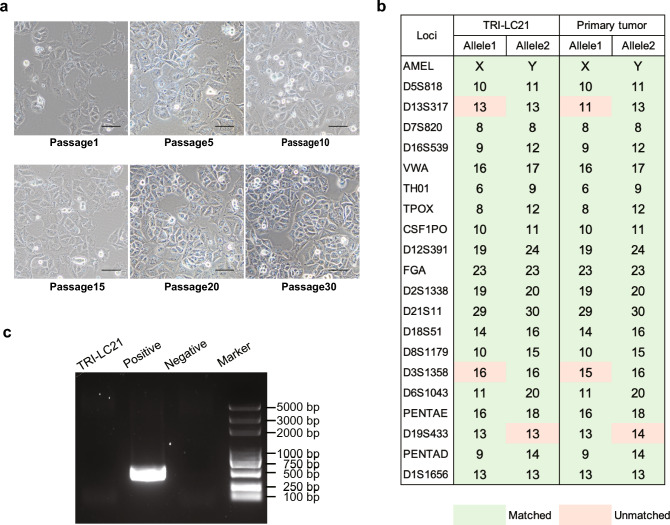
Characterization and quality control of TRI-LC21 cell line. **a** Bright-field images showing the morphology of TRI-LC21 cells at passages 1, 5, 10, 15, 20, and 30 (scale bars, 100 µm), **b** STR profiling showing a highly concordant genotype between the TRI-LC21 cell line and the corresponding primary tumor, confirming their common origin, **c** Mycoplasma detection by PCR. Lane 1, TRI-LC21 sample; lane 2, positive control; lane 3, negative control; lane 4, DNA marker

The tumor tissue was subsequently subjected to continuous in vitro culture. After more than 30 passages, a tumor cell line with stable growth characteristics was successfully established and designated as TRI-LC21. Under phase-contrast microscopy, TRI-LC21 cells exhibited adherent growth with predominantly polygonal morphology and relatively large, prominent nuclei. No obvious morphological changes were observed during serial passaging (Fig. 1a).

To confirm the genetic origin of TRI-LC21, STR profiling was performed on both the TRI-LC21 cells and the corresponding patient tumor tissue. The STR profiles were highly concordant across all 21 tested loci (92.9% match), confirming that TRI-LC21 was derived from the patient’s primary tumor, as shown in the allele comparison table (Fig. 1b) and the representative electropherograms (Supplementary Fig.S2). Furthermore, the STR profile of TRI-LC21 was queried against the DSMZ Cell Line Authentication Tool, which integrates reference datasets from ATCC, DSMZ, JCRB, and RIKEN. No matching profiles were identified among 2455 authenticated cell lines, indicating that TRI-LC21 is a novel cell line without cross-contamination. Mycoplasma testing was negative (Fig. 1c), confirming its suitability for subsequent in vitro studies.

### In vitro proliferation, tumorigenicity, and immunophenotype of TRI-LC21 cells

**Fig. 2:**
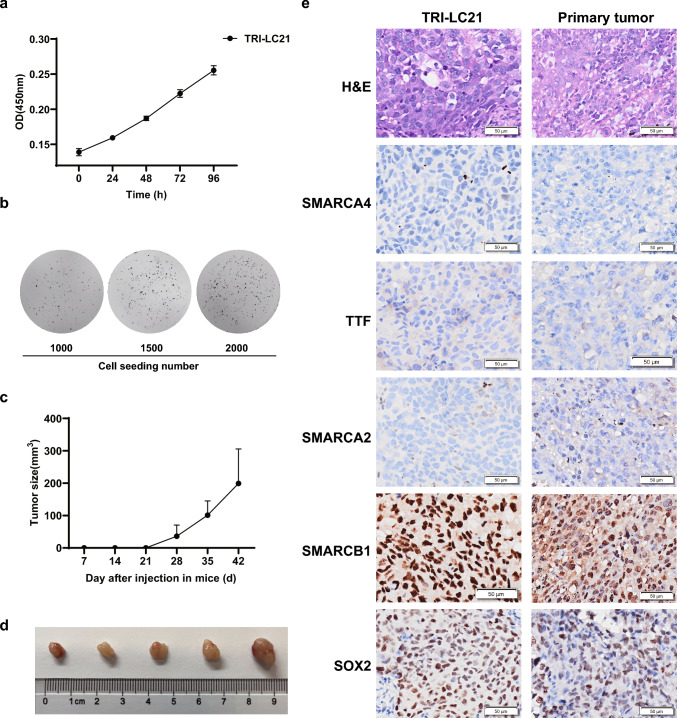
In vitro proliferation, tumorigenicity, and immunophenotype of TRI-LC21 cells. **a** Cell proliferation curve of TRI-LC21 cells monitored over a 96-h period, with measurements taken every 24 h. Data are presented as mean ± SD (*n* = 5). Error bars represent standard deviation, **b** Colony formation assay of TRI-LC21 cells plated at different densities (1000, 1500, and 2000 cells per well) and stained after 14 days, **c** Growth curve of subcutaneous xenograft tumors derived from TRI-LC21 cells in nude mice. Tumor volume was measured weekly for 6 weeks and calculated using the formula: (length × width^2^)/2. Data are presented as mean ± SD (n = 5). Error bars represent standard deviation, **d** Representative images of subcutaneous xenograft tumors excised at the end of the experiment, **e** Representative Hematoxylin and eosin (H&E) staining and immunohistochemical analysis of TRI-LC21–derived xenograft tumors and the corresponding primary tumor, including assessment of SMARCA4, TTF-1, SMARCA2, SMARCB1, and SOX2 expression. Scale bars: 50 µm

TRI-LC21 cells exhibited continuous proliferation in vitro. CCK-8 assays indicated a population doubling time of approximately 75 h (Fig. 2a). In colony formation assays, numerous colonies were observed after 14 days of culture, demonstrating a strong clonogenic capacity (Fig. 2b). To assess in vivo tumorigenicity, TRI-LC21 cells were subcutaneously injected into immunodeficient nude mice. Palpable tumor nodules appeared three weeks post-inoculation (Fig. 2c), and solid tumors were harvested at the experimental endpoint (Fig. 2d).

Comparative immunohistochemical analysis was performed on the patient’s primary tumor and TRI‑LC21 xenograft tissues to evaluate whether the cell line retains the immunophenotype of the original tumor. Both exhibited negative expression of SMARCA4, TTF-1, and SMARCA2, while SMARCB1 and SOX2 were positive. To more comprehensively characterize the xenograft, six additional markers were assessed: p40, CD56, Ki‑67, SALL4, CD34, and Vimentin. The xenograft tissues showed negative expression of p40 and CD56, with a Ki‑67 labeling index of approximately 50%. SALL4, CD34, and Vimentin were all positively expressed (Supplementary Fig.S3). These findings not only confirm the immunophenotypic concordance between TRI‑LC21 and the patient tumor, but also align with supportive diagnostic features of SMARCA4‑UT, including loss of SMARCA2 and positive expression of CD34, SOX2, and SALL4 [9, 25].

### Genomic features of the TRI-LC21 cell line

**Fig. 3:**
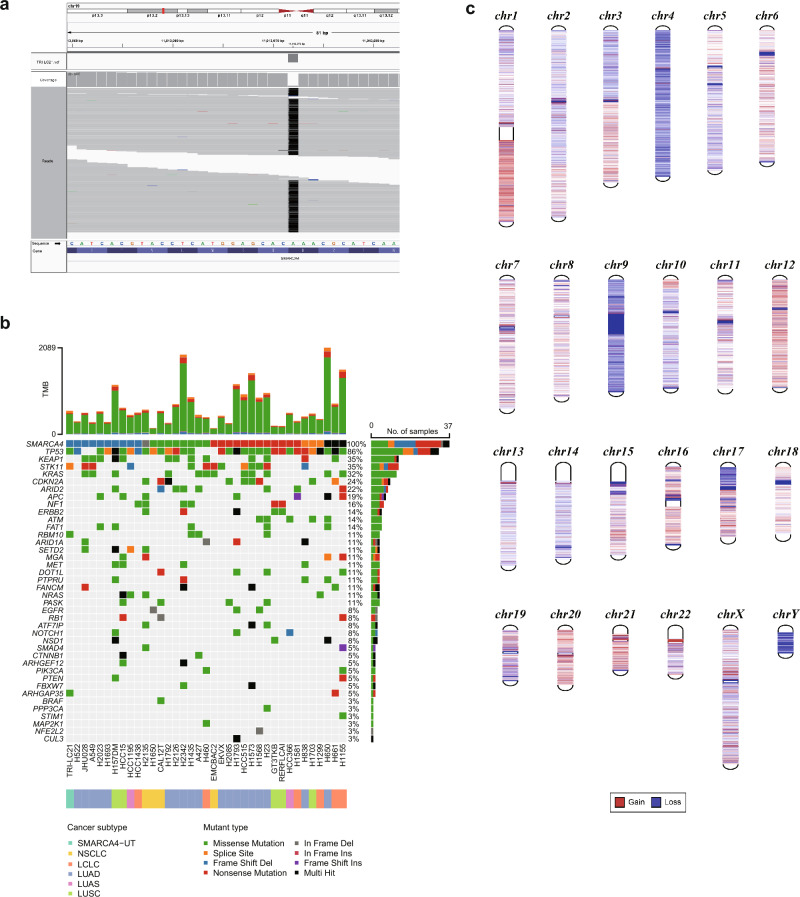
Genomic features of the TRI-LC21 cell line. **a** Integrative Genomics Viewer (IGV) visualization of the SMARCA4 frameshift deletion in TRI-LC21. The SMARCA4 c.2398delA (p.K800fs) mutation identified by whole-exome sequencing is shown, with sequencing reads supporting the deletion, **b** Mutational landscape of TRI-LC21 compared with 36 SMARCA4-mutant lung cancer cell lines. The top bar plot shows the tumor mutational burden (TMB) of each sample. The heatmap displays the mutation status of the top 40 most frequently mutated genes, with different colors representing various mutation types, as indicated in the legend. Mutation types include missense, nonsense, frameshift deletions/insertions, splice site mutations, and multi-hit events. Samples are annotated at the bottom according to histological subtype, including SMARCA4-deficient undifferentiated thoracic tumor (SMARCA4-UT), non–small cell lung cancer (NSCLC), large cell lung carcinoma (LCLC), lung adenocarcinoma (LUAD), lung adenosquamous carcinoma (LUAS), and lung squamous cell carcinoma (LUSC). The right bar plot indicates the number and frequency of mutations per gene across all samples, **c** Genome-wide copy number variation (CNV) profile of TRI-LC21, with copy number gains and losses shown in red and blue, respectively

WES was performed to characterize the genomic landscape of the TRI-LC21 cell line. A frameshift deletion in the SMARCA4 gene (c.2398delA, p.K800fs) was identified, indicating loss of SMARCA4 function. Visualization using Integrative Genomics Viewer (IGV) confirmed the presence of this mutation at the sequencing read level, with consistent support from multiple sequencing reads (Fig. 3a). Since *SMARCA4* mutations occur across different lung cancer subtypes and their biological effects are influenced by co-occurring genomic alterations [13, 26, 27], we further examined the somatic mutation profile of TRI-LC21 and compared it with 36 previously reported *SMARCA4*-mutant lung cancer cell lines. TRI-LC21 harbored concurrent mutations in *TP53*, *STK11*, *RBM10*, and *ARHGAP35* (Fig. 3b). Among these, *TP53* and *STK11* are frequently mutated tumor suppressor genes in NSCLC, particularly in tumors associated with smoking [28–31]. *RBM10* participates in RNA splicing regulation [32–34], while *ARHGAP35* is involved in Rho GTPase signaling [35, 36]. Collectively, these co-mutations are implicated in DNA damage response, cell metabolism, RNA processing, and cytoskeletal regulation [37–40].

Copy number variation (CNV) analysis revealed widespread chromosomal gains and losses in TRI-LC21 (Fig. 3c). Regions containing *NOTCH2NLC*, *ARHGEF19*, and *KCNJ16* were amplified, whereas deletions affected *CDKN2B*, *FOCAD*, and *HACD4* (Supplementary Table 1). These genes are associated with Notch signaling pathway regulation, cell migration, ion transport regulation, cell cycle control, cell adhesion and lipid metabolism [41–46]. Consistent with the CNV findings, chromosome staining showed pronounced aneuploidy, indicating substantial chromosomal instability in TRI-LC21 cells (Supplementary Fig.S4).

Overall, in the context of SMARCA4 loss, TRI-LC21 exhibits a complex somatic mutation landscape accompanied by extensive copy number alterations and chromosomal abnormalities, reflecting a high degree of genomic instability. Based on these genomic features, we subsequently performed a comprehensive transcriptomic analysis of the TRI-LC21 cell line.

### Transcriptomic features of the TRI-LC21 cell line

**Fig.4:**
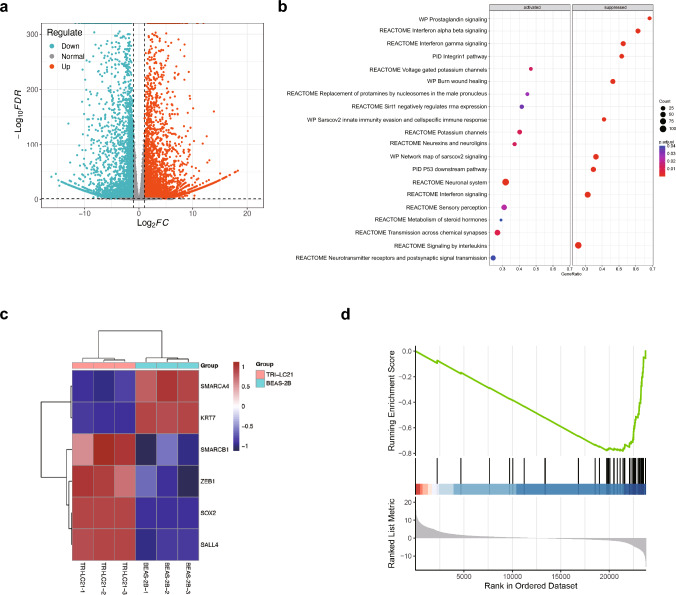
Transcriptomic features of the TRI-LC21 cell line. **a** Volcano plot showing differentially expressed genes between TRI-LC21 and BEAS-2B cells. Significantly upregulated genes are shown in red, and downregulated genes are shown in blue, **b** Gene set enrichment analysis (GSEA) dot plot showing the top enriched pathways among upregulated (left) and downregulated (right) gene sets, **c** Heatmap of key lineage and phenotypic markers in TRI-LC21 versus BEAS-2B, **d** GSEA plot showing downregulation of SMARCA4 target gene sets in TRI-LC21

Compared with the normal bronchial epithelial cell line BEAS-2B, TRI-LC21 showed extensive transcriptomic alterations, with 5593 genes upregulated and 4045 genes downregulated (Fig. 4a). Gene set enrichment analysis (GSEA) revealed that upregulated genes were predominantly enriched in neuron-related pathways and voltage-gated potassium channel activity (Fig. 4b), indicating activation of neuron-associated transcriptional programs in TRI-LC21. In contrast, downregulated genes were significantly enriched in interferon signaling, integrin-mediated pathways, and the p53 signaling cascade, suggesting attenuation of these pathways at the transcriptomic level.

Consistent with SMARCA4-deficient tumors, TRI-LC21 retained key molecular characteristics. SMARCA4 expression was markedly reduced, whereas SMARCB1 expression was preserved, distinguishing TRI-LC21 from SMARCB1-deficient malignancies [10, 47]. In parallel, expression of the epithelial marker KRT7 was decreased, while stemness-associated transcription factors SOX2 and SALL4, as well as the epithelial–mesenchymal transition regulator ZEB1, were upregulated (Fig. 4c), consistent with a poorly differentiated transcriptional profile [10, 47]. Further analysis demonstrated significant downregulation of SMARCA4 target gene sets in TRI-LC21 (Fig. 4d), supporting a broad transcriptional impact of SMARCA4 functional loss.

Collectively, these transcriptomic findings are consistent with molecular characteristics reported in SMARCA4-UT [10, 47]. When integrated with the patient’s pathological features, xenograft immunophenotype, and genomic alterations, TRI-LC21 can be classified as a SMARCA4-UT cell line [9].

## Discussion

In this study, we established and comprehensively characterized TRI-LC21, a patient-derived SMARCA4-UT cell line, providing a well-characterized in vitro model for this rare tumor.

Immunophenotypically, TRI-LC21 is defined by complete loss of SMARCA4 and lacks clear epithelial or neuroendocrine differentiation markers. Immunohistochemistry revealed negativity for TTF-1, SMARCA2, p40, and CD56, while SMARCB1, SOX2, SALL4, CD34, and Vimentin were positive, with a Ki-67 index of approximately 50%, reflecting an overall lack of defined lineage differentiation [10, 47, 48]. These features are consistent with the immunohistochemical profile of SMARCA4-UT described by the WHO 2021 classification [9], further supporting the representativeness of TRI-LC21. Previous in vitro proliferation and xenograft formation experiments demonstrated that TRI-LC21 maintains stable growth and tumorigenic capacity under both in vitro and in vivo conditions, providing an experimental foundation for studying SMARCA4-UT biology and potential therapeutic strategies.

At the molecular level, TRI-LC21 harbors a frameshift deletion in *SMARCA4* and co-occurring mutations in *TP53*, *STK11*, *RBM10*, and *ARHGAP35*, alongside widespread copy number variations and aneuploidy, indicating a highly unstable genome [10, 11]. Transcriptomic analyses revealed downregulation of epithelial-associated genes, upregulation of stemness-related transcription factors SOX2 and SALL4, as well as the cell plasticity regulator ZEB1, and overall downregulation of SMARCA4 target gene sets, collectively reflecting a poorly differentiated and lineage-plastic transcriptional state [10, 47, 48].

To date, only a limited number of SMARCA4-UT cell models have been reported, all of which have been identified through retrospective reclassification of previously established lung cancer cell lines [23, 24]. In contrast, TRI-LC21 was derived directly from primary tumor tissue, thereby preserving tumor-specific biological features that may be lost during long-term adaptation of established cell lines. This distinction may enhance its utility for studying tumor evolution and for evaluating therapeutic responses in a more physiologically relevant context.

Beyond serving as a disease model, TRI-LC21 represents a valuable preclinical platform for exploring therapeutic vulnerabilities associated with SMARCA4 deficiency. Recent studies have suggested that SMARCA4-deficient tumors may exhibit potential sensitivity to EZH2 inhibitors, ATR inhibitors, CDK4/6 inhibitors, and immunotherapeutic approaches [17, 25]. Therefore, TRI-LC21 holds promise for future drug sensitivity screening, combination therapy evaluation, and biomarker discovery. Furthermore, this model may facilitate the elucidation of acquired resistance mechanisms, thereby providing experimental evidence to optimize precision therapeutic strategies for SMARCA4-UT.

Several limitations of this study should be acknowledged. First, TRI-LC21 represents a single patient-derived model, and thus may not capture the full heterogeneity of SMARCA4-UT. Second, functional validation of specific molecular dependencies and therapeutic vulnerabilities remains to be further explored. Future studies incorporating additional patient-derived models and drug sensitivity profiling will be important to expand these findings.

In conclusion, TRI-LC21 recapitulates key immunophenotypic, genomic, and transcriptomic features of SMARCA4-UT and provides a valuable patient-derived in vitro model for mechanistic investigation and the exploration of potential therapeutic vulnerabilities in this aggressive malignancy.

## Supplementary Information

Below is the link to the electronic supplementary material.

Supplementary file1 (DOCX 16 KB)

Supplementary file2 (PDF 1143 KB)

Supplementary file3 (PDF 1571 KB)

Supplementary file4 (PDF 18071 KB)

Supplementary file5 (PDF 3602 KB)

Supplementary file6 (XLSX 542 KB)

## Data Availability

All sequencing data generated in this study are publicly available. The RNA-seq data have been deposited in the Gene Expression Omnibus (GEO) under accession number GSE328872, and the WES data have been deposited in the Sequence Read Archive (SRA) under BioProject accession number PRJNA1457423. These data will be publicly available upon publication.
