# Supplementary material for: Establishment and characterization of TRI-LC21: a novel patient-derived cell line of SMARCA4-deficient undifferentiated thoracic tumor
Source: Hum Cell. 2026 Jul 9;39(7):103. doi: 10.1007/s13577-026-01418-9 (PMC13350128; doi:10.1007/s13577-026-01418-9)
Supplement: Supplementary file 1 — Supplementary file1 (DOCX 16 KB) [file 13577_2026_1418_MOESM1_ESM.docx]

### Supplementary Fig.S1 STR profiling of the BEAS-2B cell line

1. STR locus genotyping results of BEAS-2B cells.
2. Representative electropherograms showing STR alleles across analyzed loci.

### Supplementary Fig.S2 STR profiling of TRI-LC21 and the corresponding patient tumor tissue

1. STR profile of TRI-LC21.
2. STR profile of the patient’s tumor tissue, labeled as “LC” in the figure. Short tandem repeat (STR) analysis was performed at multiple loci to authenticate the identity of TRI-LC21 and exclude cross-contamination. The STR profiles of TRI-LC21 (a) closely match those of the patient’s tumor tissue (b), demonstrating that TRI-LC21 is derived from the original tumor.

### Supplementary Fig.S3 Immunohistochemical staining of TRI-LC21 xenografts

Representative IHC images of TRI-LC21-derived xenograft tumors stained for p40, CD56, Ki‑67, SALL4, CD34, and Vimentin. Scale bars: 50 µm.

### Supplementary Fig.S4 Chromosome spread of a single TRI-LC21 cell

Representative Giemsa-stained metaphase chromosome spread of an individual TRI-LC21 cell.

### Supplementary Table 1 Copy number variation (CNV) analysis of the TRI-LC21 cell line
