# Supplementary figures and images for: Establishment and characterization of TRI-LC21: a novel patient-derived cell line of SMARCA4-deficient undifferentiated thoracic tumor
Source: Hum Cell. 2026 Jul 9;39(7):103. doi: 10.1007/s13577-026-01418-9 (PMC13350128; doi:10.1007/s13577-026-01418-9)

Supplementary Fig.S1

a

| Loci    | Allele1 | Allele2 | Allele3 |
|---------|---------|---------|---------|
| D5S818  | 12      | 13      |         |
| TH01    | 7       | 9.3     |         |
| D13S317 | 13      |         |         |
| D16S539 | 12      |         |         |
| vWA     | 17      | 18      |         |
| TPOX    | 6       | 11      |         |
| D7S820  | 10      | 13      |         |
| CSF1PO  | 9       | 12      |         |
| Amel    | X       |         |         |

b

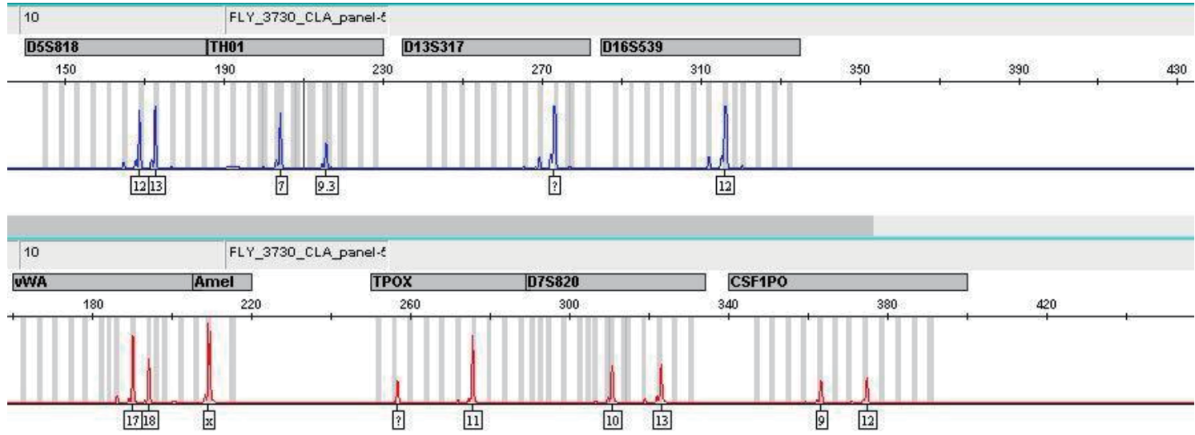

Supplement: Supplementary file 2 — Supplementary file2 (PDF 1143 KB) [file 13577_2026_1418_MOESM2_ESM.pdf]

Supplementary Fig.S2

a

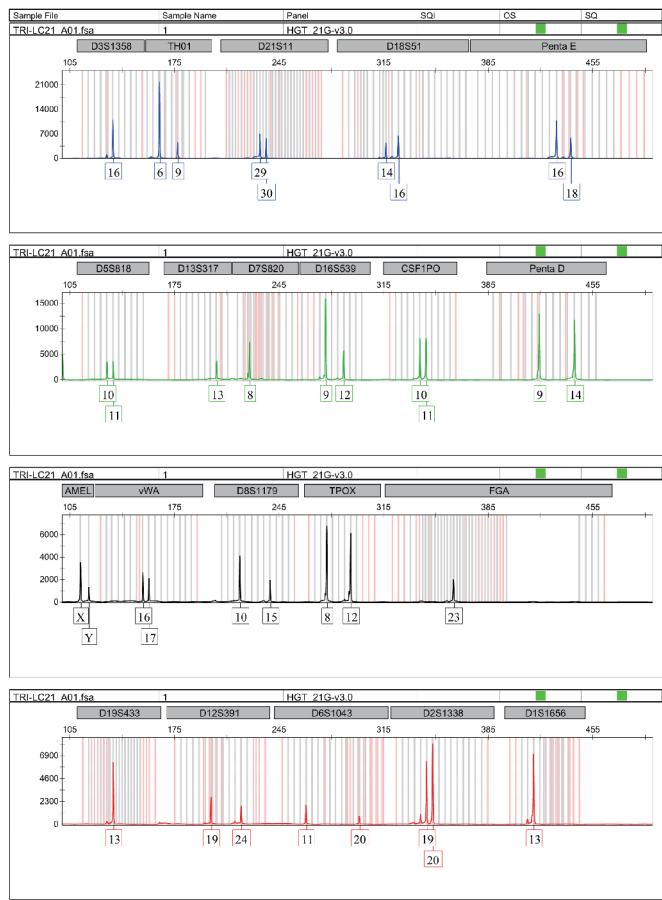

b

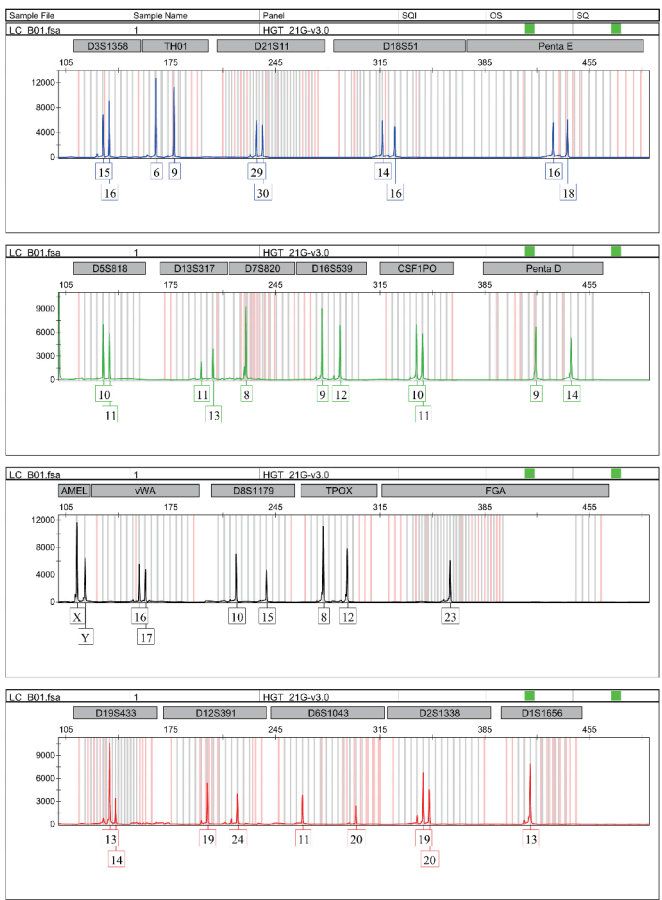

Supplement: Supplementary file 3 — Supplementary file3 (PDF 1571 KB) [file 13577_2026_1418_MOESM3_ESM.pdf]

Supplementary Fig.S3

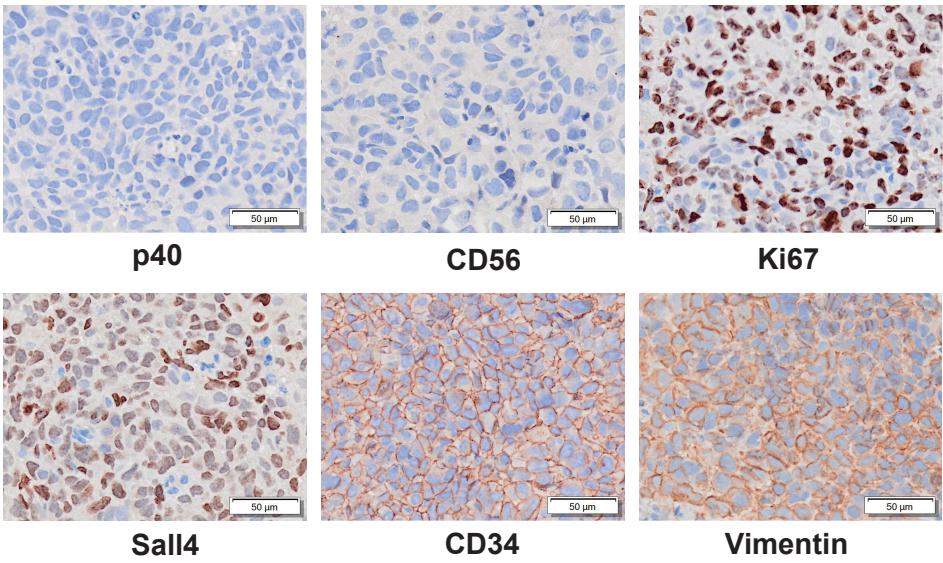

Supplement: Supplementary file 4 — Supplementary file4 (PDF 18071 KB) [file 13577_2026_1418_MOESM4_ESM.pdf]

Supplementary Fig.4

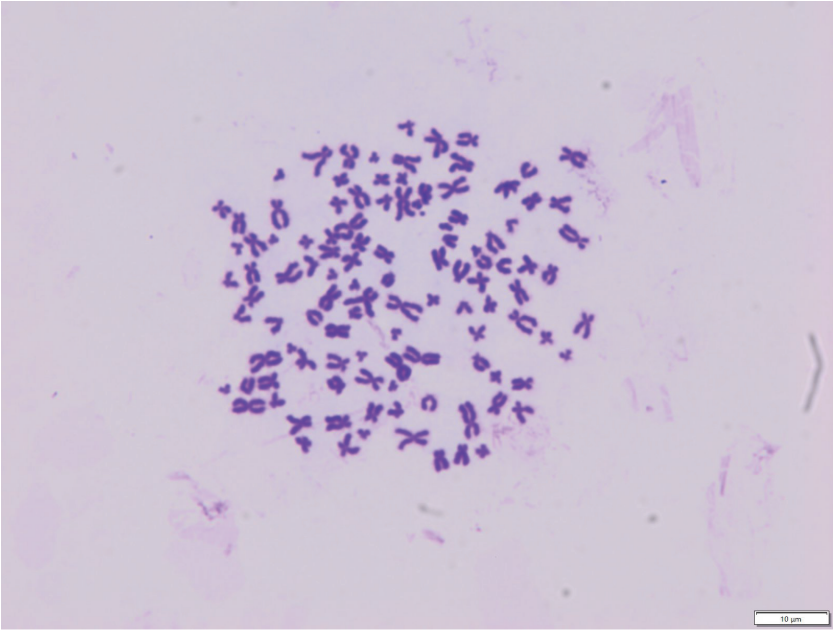

Supplement: Supplementary file 5 — Supplementary file5 (PDF 3602 KB) [file 13577_2026_1418_MOESM5_ESM.pdf]
